# Evaluation of Different Culture Media for Improvement in Bioinsecticides Production by Indigenous *Bacillus thuringiensis* and Their Application against Larvae of *Aedes aegypti*

**DOI:** 10.1155/2014/273030

**Published:** 2014-02-02

**Authors:** Patil Chandrashekhar Devidas, Borase Hemant Pandit, Patil Satish Vitthalrao

**Affiliations:** ^1^School of Life Sciences, North Maharashtra University, P.O. Box 80, Jalgaon, Maharashtra 425001, India; ^2^North Maharashtra Microbial Culture Collection Centre (NMCC), North Maharashtra University, P.O. Box 80, Jalgaon, Maharashtra 425001, India

## Abstract

Production of indigenous isolate *Bacillus thuringiensis sv2* (*Bt sv2*) was checked on conventional and nonconventional carbon and nitrogen sources in shake flasks. The effects on the production of biomass, toxin production, and spore formation capability of mosquito toxic strain were determined. Toxicity differs within the same strain depending on the growth medium. *Bt sv2* produced with pigeon pea and soya bean flour were found highly effective with LC_50_ < 4 ppm against larvae of *Aedes aegypti*. These results were comparable with bacteria produced from Luria broth as a reference medium. Cost-effective analyses have revealed that production of biopesticide from test media is highly economical. The cost of production of *Bt sv2* with soya bean flour was significantly reduced by 23-fold. The use of nonconventional sources has yielded a new knowledge in this area as the process development aspects of biomass production have been neglected as an area of research. These studies are very important from the point of media optimization for economic production of *Bacillus thuringiensis* based insecticides in mosquito control programmes.

## 1. Introduction

Microorganisms and microbial product with potential insecticidal activity can play an important role in controlling diseases by interrupting transmission mechanism by killing insect vectors at community level [1]. Worldwide efforts to screen effective entomopathogenic microorganisms for control of agriculturally and medically important insect pests have yielded many *Bacillus thuringiensis* (*Bt*) isolates with various insecticidal properties [2]. The Gram-positive bacterium *Bacillus thuringiensis* is well known for its ability to form spores and crystal proteins with insecticidal activity against a wide variety of lepidopteran, coleopteran, and dipteran insects [3]. The use of *B*. *thuringiensis* as commercial bioinsecticides was due to the remarkable ability of this bacterium to produce large quantities of insecticidal crystal proteins during large-scale fermentation. In many countries including developed ones, where mosquito borne diseases are still a serious problem, there is a need for large quantities of such microbial insecticides. Recently, more attention has been drawn to the low-cost production of *B*. *thuringiensis *which can be achieved through the optimization of culture conditions using appropriate media [4, 5]. Few published reports are available on use of low-cost ingredients for development of media for *B*. *thuringiensis* production. Agroindustrial residues and byproducts like cheese whey, soya bean milk, molasses, chicken feather waste, and paddy husk waste have been used as ingredients [6–8]. Edible seeds like mung beans (*Vigna radiata *(L.) R. Wilczek) were used as major sources of protein together with different combinations of soluble starch and/or sugarcane molasses as major carbohydrate sources for the production of delta-endotoxin [9]. Similarly, soya bean flour, groundnut cake powder, and wheat bran extract (*Triticum aestivum *L.) were separately used in large-scale production of *B. thuringiensis *bioinsecticide [10]. Media formulation and optimization are key considerations in development of bioprocesses that can produce affordable biological agents, yet limited progress has been made in this area to satisfy market opportunities for affordable commercial biological insecticide products. It has been well documented that nutrient sources like carbon, nitrogen sources, and macronutrients strongly influence the growth, spore production, and the toxicity associated with parasporal proteinaceous crystalline inclusions during sporulation and synthesis of commercially useful metabolites in *Bacillus* species [11, 12]. Commonly used nutrient sources include a wide range of peptones, extracts, and hydrolysates, many of which are expensive for industrial-scale manufacture of large-volume products and have negative market acceptance as animal byproducts [13, 14]. The study of growth medium components affecting significantly the production of biomass, toxin production, and spore formation is a step required to advance in the design of a low-cost culture medium for the efficient production of all above responses.

Therefore, in the present work, an attempt has been made to determine the effects of several conventional and nonconventional carbon and nitrogen sources on the production of biomass, toxin production, and spore formation capability of mosquito toxic strain *Bt sv*2. This study also determines the cost effectiveness of potential substrates in production of *Bt* insecticide.

## 2. Material and Methods

### 2.1. Bacteria and Growth

*Bacillus thuringiensis s* *v*2 was locally isolated in India and tested for potential mosquito toxic activity [1]. *Bt sv*2 was maintained on nutrient agar slopes (HiMedia, Mumbai) at 4°C throughout the study. The organism was grown in 50 mL of nutrient broth with shaking for 24 hrs at 30°C. This culture was further used as an inoculum (1%v/v) for a basal medium composed of 1 L water and NaCl (0.25%), Na_2_HPO_4_ (0.1%), (MgSO_4_ (0.02%), and MnCl_2_ (0.005%) and (pH 7.2)). The medium was autoclaved for 30 min at 121°C.

### 2.2. Media Preparation

Conventional and nonconventional carbon and nitrogen sources were used as test materials.

Carbon sources tested were glucose, sucrose, fructose, corn starch, mannitol, beet root pulp (*Beta vulgaris* L.), banana (*Musa paradisiaca* L. var. Grand Naine) fruit pulp, and mahua (*Madhuca indica* L.) flower extract. For carbon sources study, basal medium was supplemented with yeast extract (0.5%) and various carbon sources were incorporated so as to correspond to 10 g/L carbon concentration.

Nitrogen sources tested were soya bean flour, pigeon pea flour, yeast extract, malt extract, beef extract, egg albumin powder, casein powder, ammonium sulphate, and sodium nitrate. For nitrogen sources studies, basal medium was supplemented with glucose (10 g/L) and various nitrogen sources were incorporated at a final concentration of 0.5 g/L.

The *Bt sv*2 cells were grown for 48 hrs at 30°C with shaking at 150 rpm in a basal medium supplemented with test carbon and/or nitrogen source. The carbon and nitrogen sources added to basal media constituents were investigated to get maximum dry cell mass and to determine yield coefficient, protein content, and toxicity to mosquito larvae.

### 2.3. Preparation of Nonconventional Nutrients

#### 2.3.1. Flower Extract of Mahua

Flowers of *M*. *latifolia* L. were collected and dried in shade for 8 days at room temperature (28 ± 2°C); 100 g dry flowers were soaked in 200 mL hot distilled water (95°C) and incubated in shaker with 220 rpm at 29°C for 2 h. The extract filtered through muslin cloth was used as a source of sugar in medium.

#### 2.3.2. Banana Pulp

Banana pulp was prepared by blending the ripe banana in a mixer and reduced to puree. Distilled water was added in puree at ratio of 3 : 1 to make a final puree which could be poured.

#### 2.3.3. Beet Root

Fresh beet roots were purchased from local market. Pulp was prepared by blending the beet roots in a mixer and reduced to puree. Distilled water was added in puree at ratio of 3 : 1 to make a final puree which could be poured. This pulp was further used as source of sugar in nutrient medium.

#### 2.3.4. Pigeon Pea and Soya Bean Flour

Pigeon pea and Soya bean flour were prepared by separately grinding beans finely enough to pass through a 100-mesh.

### 2.4. Cell Mass

After fermentation was completed, two samples (50 mL) were taken from each fermenter and then centrifuged at 8000 rpm for 15 min. The supernatants were discarded and the cell pellets were lyophilized. Dry weight was calculated and expressed in grams per liter (g/L). The same sample was used for the toxicity test.

#### 2.4.1. Yield Coefficient

The yield coefficient was expressed as the ratio of carbon in the newly formed biomass to the carbon in the respective sugar source. Total carbon estimation was measured by the 3-5-dinitrosalicylic acid (DNS) modified method [15].

#### 2.4.2. Protein Determination

1 mL of culture medium was centrifuged for 10 min at 10000 g and the resulting pellets were washed twice with NaCl (1 mL) and twice with distilled water. These pellets were then suspended in 1 mL of NaOH (50 mM/L, pH 12.5) in order to solubilize protein crystals. After 2 h of incubation at 37°C, total proteins in the supernatant were measured by using the method by Bradford [16].

#### 2.4.3. Bacterial Growth

*Bt s* *v*2 was inoculated in all the test media and allowed to grow under constant agitation in the shaker (200 rpm at 30°C for 72 h). Culture samples from the respective media were drawn (2.5 mL) every 6 h, till the end of the bacterial growth (72 h). The density of culture media was measured (at 600 nm) using the UV-Vis spectrophotometer (Shimadzu, Japan). The bacterial developmental stages (vegetative to sporulation) were monitored (Labomed Microscope, India).

### 2.5. Toxicity Assay

For the laboratory trial, early fourth instars larvae of *Aedes aegypti *were collected from city area of Jalgaon (21°2′54′′N, 76°32′3′′E; elevation, 209 m). The identified larvae were kept in plastic enamel trays containing dechlorinated tap water. They were maintained, and all the experiments were carried out at 28 ± 2°C and 75–85% relative humidity under 14 : 10 light and dark cycles. Larvae were fed with a diet of finely ground brewer's yeast and dog biscuits (3 : 1) [1]. Dry cell mass produced in different media was assayed against early fourth instars larvae of *Aedes aegypti*. Bioassay was performed by dissolving lyophilized *Bt sv*2 powder in distilled water to get final different concentrations of 15, 10, 7.5, 5, 2.5, 1, and 0.5 ppm for 20 larvae in 50 mL of distilled water. Larval mortality was checked after 24 hrs of incubation. Each treatment was performed in three replicates each. In all cases, the mortality of control larvae, reared on a bacterial cell free diet (or water medium) and under the same environmental conditions as the experimental larvae, was recorded and calculated by Abbott [17] formula.

## 3. Statistical Analysis

The larvicidal activity of cell mass produced in each medium at different concentrations of 15, 10, 7.5, 5, 2.5, 1 and 0.5 ppm was subjected to probit regression analysis. The lethal concentrations in ppm (LC_50_, LC_90_) and the 95% confidence intervals of LC_50_ (upper confidence limit and lower confidence limit) were calculated. All conclusions are based on experiments that are repeated in time to ensure repeatability of results. Costs of the culture media were determined based on the ingredient prices in the western region of India. Media were compared based on their cost and potency against *Aedes aegypti *larvae.

Experimental data were analysed by one-way analysis of variance (ANOVA) using statistical software Minitab for Windows version 13. Treatment means were separated by Tukey's multiple comparison test at *α* = 0.05.

## 4. Result and Discussion

The present study aimed to maximize production of *Bt* based bioinsecticide by ensuring high level of biomass, protein, and spore production from an indigenous isolate *Bt  sv*2 with relatively cheap nutrient sources. Initially, we screened 8 carbon substrates (glucose, sucrose, fructose, starch, mannitol, banana, beet root, and mahua) based on biomass production capability at laboratory scale. Amount of biomass produced by *Bt* *sv*2 on different carbon source was found to vary (Table 1). Out of eight test carbon sources and reference medium (LB), only medium with glucose had the highest biomass yield (6.30 ± 0.03 g/L) in the basal production medium (Table 1). The second highest biomass yield (5.87 ± 0.08 g/L) was obtained in a medium with banana. Consumed sugars were determined to test rate of carbon utilization and to determine yield coefficient. Interestingly, more than 50% of initial sugars (glucose and banana) were remaining without being consumed even after prolonged fermentation studies (72 h). These results are particularly interesting because remaining residue could be revalued as economic nutrient in next fermentation batches or to fed animals. We observed that addition of sucrose, fructose, starch, mannitol, and mahua as carbon source had no significant positive effect on biomass yield. These compounds were probably not preferred by the *Bt* *sv*2 strain. On the other hand, glucose, banana, and beet root produced biomass at par with LB medium. According to our results, glucose should be added to the growth medium in order to obtain positive effects on the growth of *Bt* *sv*2, because glucose significantly stimulated biomass production. This type of response indicates that the inclusion of carbohydrates in a growth medium should be performed very carefully. Effect of these carbon sources was also analyzed by calculating their yield coefficient (*Y*); it showed that glucose is the best carbon source for biomass production (Table 1). We used glucose as an optimized carbon source in further studies considering its pure form compared to banana pulp and cost in purification of banana pulp. Our observation on banana pulp utilization is a new one in the production of *Bt* based biopesticides. Earlier, Shyam et al. [18] reported that waste ripe banana contains higher reducing sugars which helped in higher ethanol production using *Saccharomyces cerevisiae* fermentation. However, banana could also be used as an alternative to glucose after in detail factorial analysis of biomass production and cost analysis. Cell mass and product formation by microorganisms can be described quantitatively by yield coefficients expressed as the mass of cells or product formed per unit mass of substrate consumed. With the yield coefficients, the material balance equations for cells, substrate, and product can be straightforwardly formulated [19].

Since media components play a very important role in determining the yield and insecticidal activity of the spore crystal complex [20], effect of nitrogen sources on *Bt* *sv*2 was evaluated for biomass yield, toxin content, and spore production. The usable form of *Bt* based product is in the form of spores. Thus, it is important to check spore production capacity of *Bt  sv*2 on test media. It was observed that natural nitrogen sources (pigeon pea and soya bean) support more biomass and toxin production than the synthetic nitrogen sources like ammonium sulphate and sodium nitrate (Table 2).

Spore counts are known to be more accurate than dry mass for yield determinations, because dry mass is affected by suspended solids in the media. This can be seen in medium supplemented with ammonium sulphate and sodium nitrate where a dry mass was relatively obtained at par with LB medium, while the spore count was lower than expected. Medium supplemented with pigeon pea and soya bean flour had the highest yield in terms of dry mass and spores per millilitre. No strong apparent relationship was found between spore production and biomass yield. The protein concentration, an indication of toxin production from all the tested media was quantified and there was a significant difference in the production of toxins (Table 2) between the media, which demonstrates that the toxins produced from each of the media was substrate specific.

To determine a close correlation between the growth and production of bacterial agents, we measured toxin to biomass ratio. Pigeon pea containing media had the highest toxin to biomass ratio (3.78), and spore production was also high. Similar trend was observed in all media with different nitrogen sources used. Ratio of toxin produced to the biomass yield could link well with the spore production. Thus, attention should therefore be directed not only towards fermentations with high yields and/or spore production; media should be selected to obtain the high toxins per volume of biomass produced.

**Figure 1:**
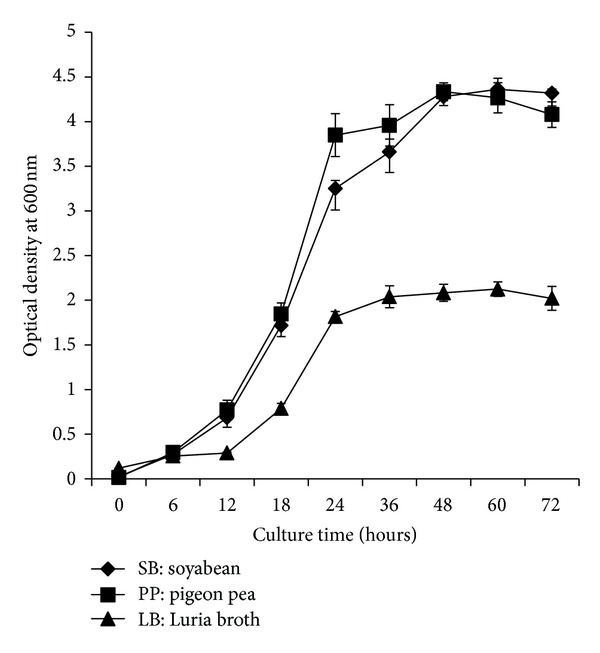
Growth pattern of *Bt sv*2 produced in different media.

In growth curve experiment, it was observed from the test culture media (pigeon pea and soya bean) that exponential phase of *Bt* *sv*2 was initiated from the sixth hour onwards (Figure 1). Rapid multiplication of bacterial cells followed by an increase in culture density was observed. This was extended up to 48 h after which the *Bt  sv*2 entered into the stationary phase of growth (48 to 72 h). Sporulation started after 48 h of growth and complete sporulation was achieved in 54 h and by 72 h the spores were found to have been released from the cells. The *Bt* *sv*2 was able to digest the nutrients from all the culture media completely by 72 h. The growth pattern of *Bt* *sv*2 in pigeon pea and soya bean medium was higher than that from LB, corroborating the results of biomass and spore production in the present study (Table 2). Similar enhanced growth pattern of *Bti* was reported with chicken feather, coconut cake, and manganese chloride based combination medium [21].

Toxicity tests (bioassays) with mosquito larvae and cost analysis were performed with *Bt sv*2 toxins produced from pigeon pea and soya bean media whereas LB was used as a reference medium.  Table 3 represents comparative toxicities of *Bt sv*2 produced from LB, pigeon pea, and soya bean media against *Aedes aegypti* larvae. Here, the effect of toxin (as measured by LC_50_ and LC_90_ values) produced from pigeon pea media was increased by more than 2-fold compared to LB media. Toxins from soya bean media (LC_50_ 3.17) had effects close to LB media (LC_50_ 4.02).

The difference in bacteria mediated larvicidal efficacy may be due to the higher production of endotoxins (cry proteins). In the pigeon pea medium there is abundance of proteins and mixed salts which made it more suitable for growth and endotoxin production. The difference in growth and toxic activities of *Bt sv*2 in different media may be due to the differences in availability of growth nutrients. It is interesting to note that the toxicity obtained at the end of the fermentation depends on the culture medium and operating conditions [22]. Boulenouar et al. [23] also observed that the different strains of the same bacteria may show different growth and toxic activities, which may be due to the differences in growth requirements of different strains. In the past, many attempts have been made to culture *Bacillus* to produce toxin at cheaper cost which has provided similar mosquito toxicity as observed in the present study [24–26].

The amount of raw materials used to prepare 1 liter of culture medium was 5 g, which is of negligible value on cost basis (Table 3). On the contrary, production on LB medium involved a cost of U.S. $ 1.61 per liter (source of price estimation was followed as per commercial preparation of fermentation media) which is 23 times more costly than pigeon pea and soya bean medium, respectively. However, transportation, electricity, and personnel cost are not included, because these expenditures are incurred commonly for both culture media. Moreover, pigeon pea and soya bean have grown in almost every part of India and most countries of the world. It has extensive usage, but storage has been its major problem as it is attacked by many pests. Damaged seeds not suitable for cultivation or as food can be used for the production of fermentation media. An alternative use, for example, in fermentation medium, may provide additional revenue to farmers particularly in India and countries that have high levels of pigeon pea or soya bean production. In the choice of materials for the production of media for *B*. *thuringiensis*, three factors should be considered: availability, cost and how well the bacterium could utilize them. Some culture media selected in the present work could represent an economical benefit for biopesticide production, because they allowed maximum biomass, spore production, and ultimately higher toxin production levels at lower cost, compared to conventional nutrient source. The media preparations examined thus separately proved adequate for cultivation of *B*. *thuringiensis* *sv*2, at least on a laboratory scale. Medium with pigeon pea, which provided the best fermentation medium for the growth and sporulation of the *Bt  sv*2 strain in this study, can be considered for further development. If these low-cost *Bt  sv*2 preparations are as successful in field trials as they are in the laboratory, they could be an important tool for use in an integrated mosquito control programme in India and other developing countries where the bioinsecticides could be produced in regional laboratories in sufficient quantities to meet instant local demands during outbreak or to preserve for later use.

## Figures and Tables

**Table 1:** Biomass production of *Bt* *sv2* in different carbon sources.

Sugar source (g/L)	SC (g/L)	Biomass (g/L)	*Y* coefficient
BC + glucose (10)	4.70 (0.02)^e^	6.30 (0.03)^a^	1.34
BC + fructose (10)	7.12 (0.04)^b^	4.30 (0.09)^c^	0.60
BC + mannitol (10)	8.02 (0.03)^a^	1.27 (0.05)^e^	0.14
BC + sucrose (10)	5.28 (0.02)^d^	3.66 (0.07)^d^	0.70
BC + starch (10)	7.93 (0.02)^a^	1.07 (0.02)^e^	0.13
BC + banana (10)	4.82 (0.03)^e^	5.87 (0.08)^b^	1.22
BC + beet root (10)	6.32 (0.08)^c^	5.10 (0.11)^b^	0.80
BC + mahua (10)	6.76 (0.04)^c^	3.61 (0.04)^d^	0.53
LB medium	—	5.40 (0.03)^b^	—

SC: Sugar consumed at the end of fermentation; initial sugar concentration used in each test medium = 10 gm/L; BC: basal medium for carbon source study (NaCl 2.5 gm/L + Na_2_HPO_4 _1 gm/L + MgSO_4 _0.2 gm/L + MnCl_2 _0.05 gm/L + yeast extract 5 gm/L). Yield (*Y*) coefficient expressed as the ratio of carbon in the newly formed biomass to the carbon in the respective sugar source.

Data are presented as mean (SD).

Means within a given column followed by the same letter are not significantly different, Tukey's MRT (*α* = 0.05), *P* < 0.0001.

**Table 2:** Comparative analysis of *Bt* *sv2* production in different nitrogen sources.

Nitrogen source (g/L)	Biomass yield (g/L)	Toxin content (protein mg/L)	Spores (CFU/mL)	Toxin/biomass (mg/g)
BN + ammonium sulphate (5)	5.44 (0.05)^c^	16.0 (0.20)^c^	7.30 × 10^7^	2.94
BN + sodium nitrate (5)	4.90 (0.10)^d^	14.2 (0.30)^e^	9.10 × 10^6^	2.89
BN + egg albumin (5)	4.96 (0.50)^d^	15.5 (0.20)^c^	1.50 × 10^7^	3.12
BN + beef extract (5)	4.70 (0.03)^d^	11.3 (0.50)^d^	1.40 × 10^6^	2.40
BN + casein (5)	6.67 (0.05)^b^	18.4 (0.60)^b^	1.69 × 10^8^	2.75
BN + malt extract (5)	5.81 (0.06)^c^	15.4 (0.80)^c^	2.40 × 10^8^	2.65
BN + soybean (5)	6.54 (0.04)^b^	19.7 (0.20)^b^	1.12 × 10^9^	3.01
BN + pigeon pea (5)	7.45 (0.09)^a^	28.2 (0.70)^a^	2.24 × 10^9^	3.78
BN + yeast extract (5)	6.26 (0.08)^b^	16.7 (0.40)^c^	9.10 × 10^8^	2.66
LB medium (25)	5.40 (0.02)^c^	18.8 (0.20)^b^	1.32 × 10^9^	3.48

BN: Basal medium for nitrogen source study (NaCl 2.5 gm/L + Na_2_HPO_4 _1 gm/L + MgSO_4 _0.2 gm/L + MnCl_2 _0.05 gm/L + glucose 10 gm/L);

data are presented as mean (SD). Means within a given column followed by the same letter are not significantly different, Tukey's MRT (*α* = 0.05), *P* < 0.0001.

**Table 3:** Toxic effect of *Bt* *sv2* on different media against IVth instars larvae of *Aedes aegypti* and comparative cost analysis for production of toxin.

Culture media (g/L)	LC_50_ ± SE (LCL-UCL)	LC_90_ ± SE (LCL-UCL)	Regression equation	Cost per liter in USD	Net difference* (in ratio)
LB medium (25)	4.02 ± 0.26 (3.57–4.65)	7.59 ± 0.64 (6.56–9.25)	*Y* = 0.438 + 2.37*X*	1.61	—
BN + pigeon pea (5)	1.57 ± 0.09 (1.37–1.76)	3.20 ± 0.17 (2.89–3.62)	*Y* = 3.66 + 3.69*X*	0.07	23
BN + soyabean (5)	3.17 ± 0.16 (2.86–3.54)	5.94 ± 0.37 (5.31–6.85)	*Y* = 0.167 + 3.12*X*	0.07	23

LC_50_: lethal concentration that kills 50% of the exposed larvae, LC_90_: lethal concentration that kills 90% of the exposed larvae, SE: standard error, and LCL-UCL: 95% upper and lower fiducial limits. LC_50_ and LC_90 _expressed in ppm.

*Ratio of cost of LB medium required for preparation of 1 L medium with the test medium.
